# A robust planning approach for respiratory motion in accelerated partial breast irradiation using volumetric modulated arc therapy

**DOI:** 10.1093/jrr/rraf011

**Published:** 2025-03-10

**Authors:** Ryohei Yamauchi, Fumihiro Tomita, Satoshi Ishikura

**Affiliations:** Department of Radiation Oncology, St. Luke’s International Hospital, 9-1 Akashi-cho, Chuo-ku, Tokyo 104-8560, Japan; Department of Radiation Oncology, St. Luke’s International Hospital, 9-1 Akashi-cho, Chuo-ku, Tokyo 104-8560, Japan; Department of Radiation Oncology, St. Luke’s International Hospital, 9-1 Akashi-cho, Chuo-ku, Tokyo 104-8560, Japan

**Keywords:** breast cancer, accelerated partial breast irradiation, intensity modulated radiation therapy, respiratory motion

## Abstract

Accelerated partial breast irradiation (APBI) is an alternative treatment for early-stage breast cancer. This study aimed to evaluate the effectiveness of the virtual bolus (VB) method and robust planning against respiratory motion in volumetric modulated arc therapy (VMAT)-APBI. VMAT plans were generated with 30 Gy in 5 fractions for 16 patients. Four treatment plans were developed and compared: a standard optimization (SO) plan without robust methods, a pseudo-skin flash strategy using a 5 mm VB (with densities of 0.4 and 1.0 g/cm^3^, VB04 and VB10), and a robust optimization (RO) plan to minimize penalties in worst-case scenarios. The isocenter was shifted 1–5 mm in each translational direction in robust analysis, and perturbed dose calculations were performed. All dose constraints for the target in SO and VB plans were within acceptable limits, but the dose evaluation volume V_95%_ in the RO plan was lower than in other plans (*P* < 0.05). The clinical target volume V_95%_ of the RO plan was significantly higher than in VB04 and VB10 (*P* < 0.05). The RO plan showed the best performance for organs at risk, followed by SO and VB plans, which resulted in higher doses. The RO plan exhibited the smallest change (±2%) in dose distribution due to respiratory motion. By contrast, the SO plan lacked robustness owing to absence of sufficient fluence in the air surrounding the planning target volume outside of the skin surface. The RO plan offers superior target coverage, maximum dose, and robustness compared to SO and VB methods.

## INTRODUCTION

Breast cancer is one of the most prevalent malignancies affecting women worldwide, necessitating continuous advancements in treatment modalities to improve patient outcomes and quality of life. Accelerated partial breast irradiation (APBI) has emerged as a promising alternative to traditional whole breast irradiation (WBI) for selected patients with early-stage breast cancer. APBI focuses on radiation on the lumpectomy cavity or tumor bed over a shorter period, thereby reducing treatment duration and potentially minimizing radiation exposure to surrounding healthy tissues. Several trials are currently investigating the potential of external beam APBI to reduce the dose to normal tissues and improve cosmesis due to the decreased volume of treated breast tissue [1–5]. Recent trials have demonstrated the dosimetric advantages of APBI combined with intensity modulated radiation therapy (IMRT) or volumetric modulated arc therapy (VMAT) [6–10]. VMAT provides comparable dose distribution for Asian women of small physique and breast size [11]. However, studies have reported that when the surgical cavity is large relative to the breast size, radiation dose to the normal mammary glands may be excessive [12, 13].

When VMAT is applied to patients with breast cancer, factors such as respiratory motion, tissue swelling, and deformity should be considered. As a countermeasure against these uncertainties, the ‘pseudo-skin flash’ strategy using a virtual bolus (VB) proposed by Nicolini *et al.* is widely used for breast IMRT [14]. However, most previous reports have focused on WBI and postmastectomy radiation therapy (PMRT), and few reports have focused on APBI [15–17]. Lizondo *et al.* reported that a VB thickness of +5 mm planning target volume (PTV) margin and a VB HU value of −500 are appropriate settings for breast IMRT to minimize dose normalization and ensure robustness rather than the previously used VB with a density equivalent to water (1.0 g/cm^3^) [18]. However, the usefulness of using the VB method in APBI is unclear, and the effect of VB density has not been reported. The advantage of the VB method is that it can be used at any facility. Conversely, it is complicated by the need to utilize multiple computed tomography (CT) data sets in the treatment planning process. Furthermore, it is commonly reported that the dose distribution is degraded when final calculations are performed on CT data sets with VB removed [18–20].

Robust planning or robust optimization (RO), an optimization method that considers setup errors, has received much attention and is robust against uncertainty [21]. Miyasaka *et al.* found that robust planning is robust to respiratory motion in patients with PMRT compared to the VB method [20]. However, the effectiveness of robust planning for respiratory motion in APBI remains unclear.

Asian women are generally smaller in stature and breast size, and APBI for such patients results in partial PTV spread outside the breast when PTV is extended. It is unclear how best to account for and optimize PTV extension outside the breast in treatment planning and robustness to respiratory motion. This study aimed to evaluate the effectiveness of the VB method and robust planning for respiratory motion in APBI with VMAT.

## METHODS AND MATERIALS

### Planning study for volumetric modulated arc therapy-accelerated partial breast irradiation planning

#### Study population

This study pooled data from 16 patients of Japanese ethnicity with early-stage breast cancer (8 with right breast cancer and 8 with left breast cancer) who were treated at our institution. The tumor sites of the patients with breast cancer were evenly distributed, with two patients in each quadrant. The study protocol was approved by our institutional review board (22-R009). This was a simulated treatment planning study solely conducted for research purposes and did not influence the clinical care of any patient.

#### Delineation for target and organs at risk

CT simulation was performed with the patient in the supine position, with both arms raised above the head via wing-board immobilization (CIVCO Radiotherapy, Orange City, IA, USA). CT images (2.0 mm slice thickness) were obtained using the SOMATOM Confidence RT Pro (Siemens Healthcare, Erlangen, Germany).

An experienced radiation oncologist delineated the clinical target volume (CTV) for this study using a surgical titanium clip as a reference. PTV was defined by adding 5 mm margins to CTV. A dose evaluation volume (PTV_Eval) was created to evaluate dose coverage at PTV, which excluded 3 mm cropped from the skin surface. The organs at risk (OARs), including the heart, bilateral breasts, bilateral lungs, and thyroid, were delineated by radiation oncologists or medical physicists. Body_outside_PTV was defined by subtracting PTV from the body structure. These structure definitions agreed with the RTOG Breast Cancer Atlas and an ASTRO clinical practice guideline [22, 23].

#### Volumetric modulated arc therapy-accelerated partial breast irradiation planning

Four plans with different planning approaches were generated using a radiation treatment planning system (RayStation version 10ASP1; RaySearch Laboratories, Stockholm, Sweden). All plans were generated by two partial arc VMAT plans. For left-sided plans, the starting and ending gantry angles were set between 155° and 285°, and the arc angle was within 210°. The angles were set between 70° and 210° for right-sided plans, and the arc angle was within 210°. Dose calculation was performed using a collapsed-cone convolution algorithm with heterogeneity correction and a constant 2 mm calculation grid size. The prescribed dose was 30 Gy in five fractions. The dose received by at least 50% of PTV_Eval volume (D_50%_) was set to 30 Gy. A 6 MV flattening-filter-free beam (TrueBeam; Varian Medical Systems, Palo Alto, CA, USA) was employed for all plans, with the couch angle held static at 0° and the control point spacing set to 2°.

First, a standard optimization (SO) plan without a robust approach for evaluation was designed to prescribe doses for PTV_Eval. Optimization was performed on the target, excluding the outer 3 mm from the skin surface to avoid absorbed dose compensation in the build-up region by the optimizer. Second, the pseudo-skin flash strategy using a VB was applied to create robust radiotherapy planning by reducing positioning and tissue deformation uncertainties. A VB of 5 mm thickness with a mass density of 1.0 g/cm^3^ was created by adding 5 mm to PTV. The plan was designed to cover the prescribed dose for the whole PTV, including the VB area outside the body contour. Dose distribution was recalculated without the VB, whereas all other settings (such as MUs and MLC segments) were identical. Third, using a similar VB10 plan method, a VB of 5 mm thickness with a mass density of 0.4 g/cm^3^ was created. This VB setting condition was established based on the report of Lizondo *et al.* [18]. The treatment planning method was the same as VB10, except for VB density. Fourth, the integrated RO tool was employed in RayStation. The RO tool employs a minimax optimization method that optimizes to minimize the penalty in worst-case scenarios. The minimax approach avoids this excessive conservatism by considering only physically feasible scenarios, evaluating the overall objective function across multiple scenarios, and selecting the least favorable of these [21]. Herein, a value of 5 mm in each direction was entered as the uncertainty set in the optimization settings and applied to CTV. Therefore, seven scenarios were considered, including no shift and patient position uncertainty in six directions for one plan. The plan was designed to cover the prescribed dose for PTV_Eval. These plans were named SO, VB10, VB04, and RO, respectively.

The plan optimization for all planning approaches was conducted using target and OAR dose constraints based on an ASTRO clinical practice guideline [23]. However, because it sets an optimization function for CTV in the RO method, the V_95%_ PTV_Eval constraint could not be satisfied; therefore, it was excluded from the dose constraints to be achieved. No restrictions were imposed on planning parameters, such as optimization settings (e.g. objectives, constraints, and the number of iterations) or monitor units (MUs), across different planning approaches. Regardless of the planning method, dose normalization was in principle set to achieve a prescribed PTV_Eval dose of 30 Gy. However, in RayStation, dose normalization was not feasible when dynamic mechanical constraints, such as exceeding the maximum gantry speed, were encountered. This issue occurred when normalization was attempted in the dose reduction direction. Therefore, no plan failed to satisfy the minimum dose constraint of 30 Gy.

#### Plan evaluation

The treatment plans created by the four optimization methods presented in the previous subsection were compared. The evaluated dose–volume histogram (DVH) parameters were the following ASTRO clinical practice guideline dose constraints: PTV_Eval V_95%_, V_105%_, D_50%_, D_max_; heart D_mean_, V_5%_, V_15%_; ipsilateral breast V_95%_, V_50%_; and ipsilateral lung V_30%_. CTV V_95%_, V_105%_, D_50%_, and ipsilateral lung V_10%_, V_50%_ were collected for dose distribution evaluation.

To evaluate the complexity of the plans among the planning approaches, the MUs, estimated beam-on time, average beam’s eye view area, aperture area variability, leaf sequence variability, and modulation complexity score for VMAT (MCSv) were calculated. These calculations were performed using a free software package (Universal Complexity Metrics Extractor) with MATLAB [24]. Complexity metrics were also compared between left- and right-sided breast cancer cases to evaluate the impact of heart dose constraints on the optimization process.

### Perturbed dose analysis

The perturbed dose calculation tool integrated into RayStation was employed to assess the robustness of the four planning approaches for each SO, VB04, VB10, and RO plan. This tool generated perturbed dose distributions by shifting the isocenter along the translational three-axis. Isocenter shifts of 1, 2, 3, 4, and 5 mm were applied, corresponding to the CTV-PTV margin or RO position uncertainty. These specific isocenter shifts were selected to evaluate whether the VB or RO method effectively accounts for interfraction and intrafraction uncertainties. The dosimetric parameters in CTV V_95%_, V_105%_, D_50%_, heart D_mean_, and ipsilateral lung V_30%_ were compared between the original and perturbed dose plans.

The relationship between patient-specific radiation conditions, including tumor site and CTV shape (sphericity), and the perturbed dose when shifted 5 mm in each direction was evaluated. Sphericity, a shape index, was calculated in MATLAB as follows:


(1) \begin{equation*} Sphericity=\frac{\pi^{1/3}\ {(6V)}^{2/3}}{A}, \end{equation*}

where *A* and *V* are the surface area and volume, respectively. These values were calculated using triangle mesh analysis, with the surface area and volume derived from summing and integrating over the triangle mesh generated from the vertex coordinates of the contour.

### Statistical analysis

For statistical analysis, EZR version 1.37 (Saitama Medical Center, Jichi Medical University, Saitama, Japan), a graphical user interface for R (R Foundation for Statistical Computing, Vienna, Austria), was employed [25]. A Friedman test with Bonferroni’s correction and Mann–Whitney U test was conducted, and *P* < 0.05 was considered statistically significant.

## RESULTS

### Evaluation of the treatment plan

The mean ± standard deviation (SD) age of all patients was 54.4 ± 9.9 years, and the mean ± SD body mass index was 25.3 ± 6.0 kg/m^2^. The mean ± SD volume of CTV, PTV, PTV_Eval, and ipsilateral breast for all patients were 68.0 ± 21.9, 124.2 ± 27.6, 94.3 ± 32.8, and 632.4 ± 292.7 cm^3^, respectively.

**Fig. 1:**
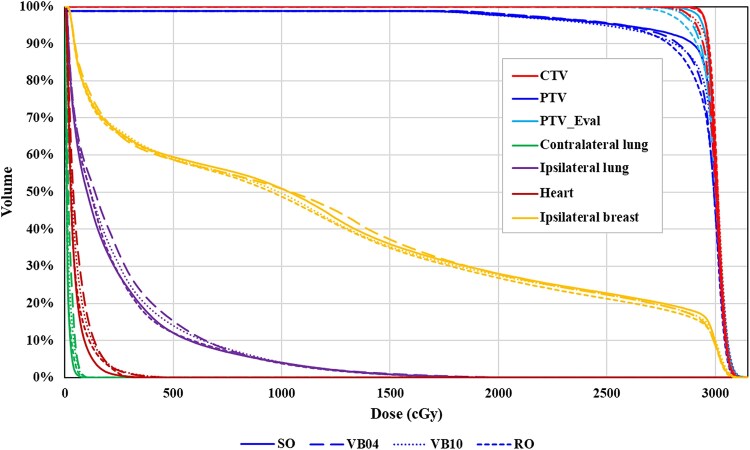
An example of DVH of a patient for four different optimization methods. Targets and organs in plans developed with different optimization methods (solid, dashed, dotted, and small dashed lines are SO, VB04, VB10, and RO) are paired in the same color. Abbreviations: SO, standard optimization plan; VB04, virtual bolus plan (*d* = 0.4 g/cm ^3^); VB10, virtual bolus plan (*d* = 1.0 g/cm ^3^); RO, robust optimization plan.

Figure 1 shows an example of DVH for each VMAT-APBI plan. Table 1 summarizes the dosimetric parameters of target and normal tissues. The mean ± SD of PTV_Eval V_95%_ for SO, VB04, VB10, and RO plans was 98.1% ± 1.3%, 97.7% ± 0.8%, 95.7% ± 2.0%, and 92.4% ± 2.0%, respectively. The mean ± SD of CTV V_95%_ for SO, VB04, VB10, and RO plans was 99.3% ± 0.7%, 98.6% ± 0.9%, 96.6% ± 2.3%, and 99.5% ± 0.5%, respectively. Although all dose constraints for the target in SO and VB plans (VB04 and VB10) were within acceptable limits, PTV_Eval V_95%_ in the RO plan was lower than the others (*P* < 0.05) and did not satisfy the ideal values suggested in the guidelines. In contrast, the CTV V_95%_ of the RO plan was significantly higher than VB04 and VB10 (*P* < 0.05). The mean ± SD of PTV_Eval V_105%_ for SO, VB04, VB10, and RO plans was 0.1% ± 0.2%, 0.1% ± 0.1%, 1.1% ± 1.3%, and 0.2% ± 0.2%, respectively. The V_105%_ for VB10 was the largest, with a difference of ~1.0%. Overall, the SO plan had better V_95%_ and V_105%_ of CTV and PTV_Eval than the others, and the VB10 plan had worse V_95%_ and V_105%_ than the others.

**Table 1:** Dose constraints for target and normal tissues and dosimetric parameters for the 16 plans

Variables	Objectives	Planning approaches (Mean ± SD)				*P* value
		SO	VB04	VB10	RO	
**CTV**						
V_95%_ (%)	≧95%	99.3 ± 0.7	98.6 ± 0.9	96.6 ± 2.3	99.5 ± 0.5	b,d,e,f
V_105%_ (%)	≦5%	0.1 ± 0.2	0.1 ± 0.1	1.1 ± 1.5	0.2 ± 0.3	b,d
D_50%_ (cGy)	≧3000 cGy	3006.4 ± 5.0	3005.6 ± 4.2	3025.8 ± 14.3	3014.6 ± 5.4	b,c,d,e,f
**PTV_Eval**						
V_95%_ (%)	≧95%	98.1 ± 1.3	97.7 ± 0.8	95.7 ± 2.0	92.4 ± 2.0	b,c,d,e,f
V_105%_ (%)	≦5%	0.1 ± 0.2	0.1 ± 0.1	1.1 ± 1.3	0.2 ± 0.2	b,d
D_50%_ (cGy)	≧3000 cGy	3003.9 ± 3.9	3002.3 ± 3.8	3020.2 ± 13.9	3002.2 ± 3.2	b,d,f
D_max_ (cGy)	≦3300 cGy	3158.6 ± 19.4	3154.8 ± 20.1	3180.5 ± 25.3	3156.2 ± 28.5	b,d
**Heart (right)**						
D_Mean_ (cGy)	<70 cGy	30.8 ± 8.9	41.7 ± 9.9	40.5 ± 13.8	31.4 ± 9.6	
V_1.5 Gy_ (%)	≦5%	0.7 ± 1.5	1.8 ± 2.4	1.6 ± 2.5	0.7 ± 1.4	
**Heart (left)**						
D_Mean_ (cGy)	<150 cGy	43.2 ± 28.9	47.9 ± 22.1	45.0 ± 23.6	41.7 ± 23.4	a,b,e,f
V_4.5 Gy_ (%)	≦5%	0.5 ± 1.1	0.5 ± 0.9	0.5 ± 1.1	0.5 ± 0.9	
**Contralateral breast**						
D_max_ (cGy)	≦90 cGy	106.4 ± 57.0	118.8 ± 70.7	109.2 ± 61.6	101.6 ± 53.3	a,c,e
**Ipsilateral breast**						
V_50%_ (%)	≦50%	34.9 ± 9.5	34.4 ± 9.5	35.3 ± 10.4	33.2 ± 9.1	c,e,f
V_95%_ (%)	≦25%	17.6 ± 4.9	17.4 ± 5.0	16.8 ± 4.5	16.0 ± 4.5	b,c,d,e,f
**Contralateral lung**						
V_10%_ (%)	≦5%	0.0 ± 0.0	0.0 ± 0.1	0.0 ± 0.1	0.0 ± 0.0	
**Ipsilateral lung**						
V_10%_ (%)	≦30%	21.6 ± 5.1	24.9 ± 5.2	23.9 ± 5.2	21.9 ± 4.8	a,b,e,f
V_30%_ (%)	≦10%	5.3 ± 2.3	5.8 ± 2.3	5.8 ± 2.3	5.0 ± 2.1	a,b,c,e,f
V_50%_ (%)	≦3%	1.6 ± 1.1	1.7 ± 1.2	1.7 ± 1.2	1.4 ± 1.0	c,e,f
**Thyroid**						
D_max_ (cGy)	≦90 cGy	8.5 ± 18.3	9.7 ± 20.7	9.5 ± 20.2	8.4 ± 17.7	a,b,e,f
**Body outside PTV**						
V_107%_ (cc)	≦2 cc	0.0 ± 0.0	0.0 ± 0.0	0.0 ± 0.0	0.0 ± 0.0	
D_max_ (cGy)	≦3300 cGy	3099.9 ± 29.6	3075.7 ± 19.9	3085.5 ± 22.2	3058.2 ± 55.5	a,c

Abbreviations: CTV, clinical target volume; PTV, planning target volume; SD, standard deviation; SO, standard optimization plan; VB04, virtual bolus plan (*d* = 0.4 g/cm^3^); VB10, virtual bolus plan (*d* = 1.0 g/cm^3^); RO, robust optimization plan.

Notes: A Friedman test with Bonferroni’s correction was performed, and statistically significant pairs (*P* < 0.05) were indicated by symbols as follows: ^a^SO vs. VB04; ^b^SO vs. VB10; ^c^SO vs. RO; ^d^VB04 vs. VB10; ^e^VB04 vs. RO; ^f^VB10 vs. RO.

Regarding DVH parameters for OAR, the RO plan showed the best performance, followed by the SO plan and VB methods with higher doses. The RO plan had significantly lower doses than VB04 and VB10 plans in heart D_mean_, contralateral breast D_max_ (VB04 only), ipsilateral breast V_50%_, V_95%_, ipsilateral lung V_10%_, V_30%_, V_50%_, and thyroid D_max_ (*P* < 0.05). For contralateral breast D_max_, ipsilateral breast V_50%_, V_95%_, and ipsilateral lung V_30%_, the RO plan had significantly lower doses than the SO plan (*P* < 0.05). The SO plan had significantly lower doses than the VB method for heart D_mean_, ipsilateral lung V_10%_, V_30%_, and thyroid D_max_ (*P* < 0.05). The contralateral breast D_max_ did not satisfy the constraints in the medial tumor case, regardless of the plan approach.

Regarding VB density differences, the VB04 plan significantly exceeded V_95%_ and V_105%_ targets; however, the VB10 plan was comparable to or better than the VB04 plan for the OAR dose.

**Fig. 2:**
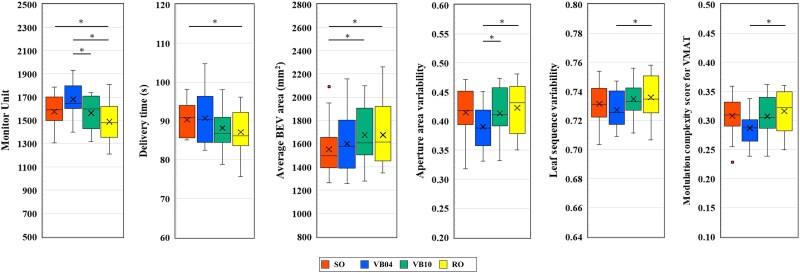
Plan complexities of each planning approach. Abbreviations: SO, standard optimization plan; VB04, virtual bolus plan (*d* = 0.4 g/cm ^3^); VB10, virtual bolus plan (*d* = 1.0 g/cm ^3^); RO, robust optimization plan; BEV, beam’s eye view; VMAT, volumetric modulated arc therapy. * *P* < 0.05.

Table 2 and Fig. 2 show the plan complexities of each planning approach. The mean ± SD MUs of SO, VB04, VB10, and RO were 1576.1 ± 140.3, 1682.5 ± 146.3, 1569.4 ± 147, and 1490.2 ± 161.7 MUs, respectively. The MUs of the RO plan were significantly lower than those of the other plans (*P* < 0.05). The beam-on times were 90.1 ± 4.4, 90.5 ± 6.8, 88.6 ± 5.2, and 87.1 ± 5.5 s, respectively, with a significant difference between SO and RO plans. The mean ± SD MCSv of SO, VB04, VB10, and RO were 0.308 ± 0.034, 0.287 ± 0.03, 0.307 ± 0.035, and 0.316 ± 0.036, respectively. The MCSv of the RO plan was significantly higher than that of the VB04 plan (*P* < 0.05). No significant differences in complexity metrics were observed between left- and right-sided breast cancer cases regardless of the planning approach (data not shown).

**Table 2:** Summary of plan complexities for the 16 plans

Variables	Planning approaches (Mean ± SD)				*P* value
	SO	VB04	VB10	RO	
Monitor units	1576.1 ± 140.3	1682.5 ± 146.3	1569.4 ± 147	1490.2 ± 161.7	c,d,e
Beam-on time (s)	90.1 ± 4.4	90.5 ± 6.8	88.6 ± 5.2	87.1 ± 5.5	c
Average BEV area (mm^2^)	1551.1 ± 231.1	1600.5 ± 269.2	1675.7 ± 251.2	1676 ± 267.4	b, c
Aperture area variability	0.41 ± 0.04	0.39 ± 0.04	0.41 ± 0.04	0.42 ± 0.04	d, e
Leaf sequence variability	0.73 ± 0.01	0.73 ± 0.01	0.73 ± 0.01	0.74 ± 0.01	e
Modulation complexity score for VMAT	0.308 ± 0.034	0.287 ± 0.030	0.307 ± 0.035	0.316 ± 0.036	e

Abbreviations: SD, standard deviation; SO, standard optimization plan; VB04, virtual bolus plan (*d* = 0.4 g/cm^3^); VB10, virtual bolus plan (*d* = 1.0 g/cm^3^); RO, robust optimization plan; BEV, beam’s eye view; VMAT, volumetric arc modulation therapy.

Notes: A Friedman test with Bonferroni’s correction was performed, and statistically significant pairs (*P* < 0.05) were indicated by symbols as follows: ^a^SO vs. VB04; ^b^SO vs. VB10; ^c^SO vs. RO; ^d^VB04 vs. VB10; ^e^VB04 vs. RO; ^f^VB10 vs. RO.

### Evaluation of the effects of respiratory motion

**Fig. 3:**
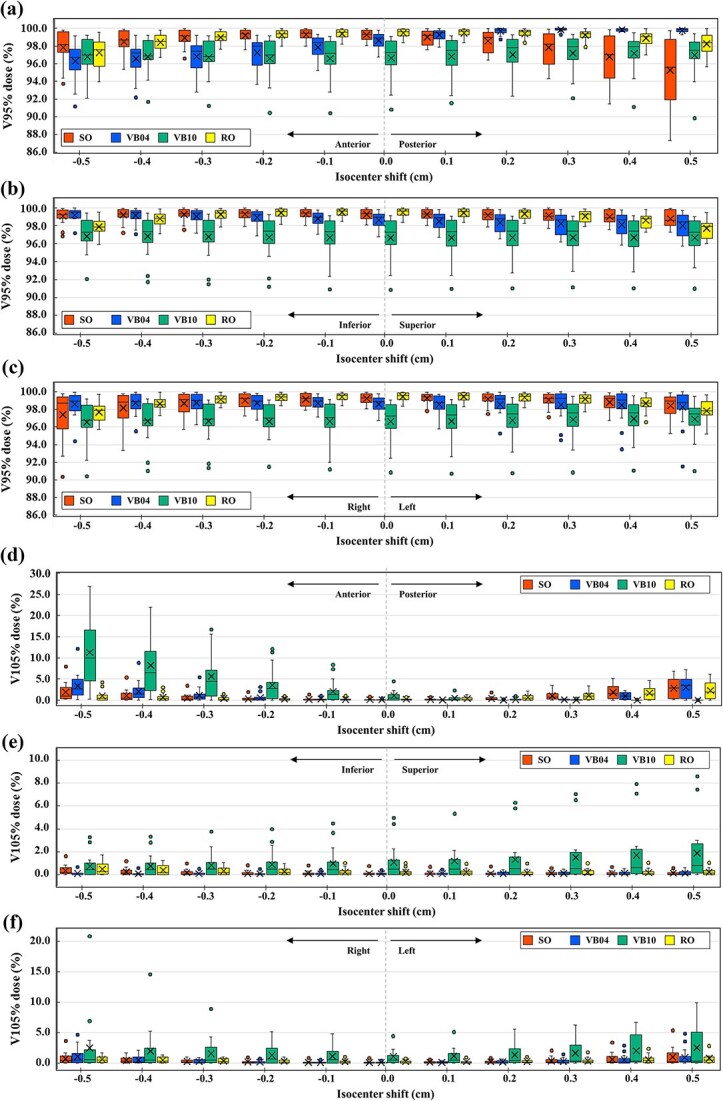
Comparison of box plots of CTV V_95%_ (a: anterior–posterior, b: inferior–superior, c: right–left) and V_105%_ (d: anterior–posterior, e: inferior–superior, f: right–left) for four different optimization methods in each isocenter shift direction.

**Fig. 4:**
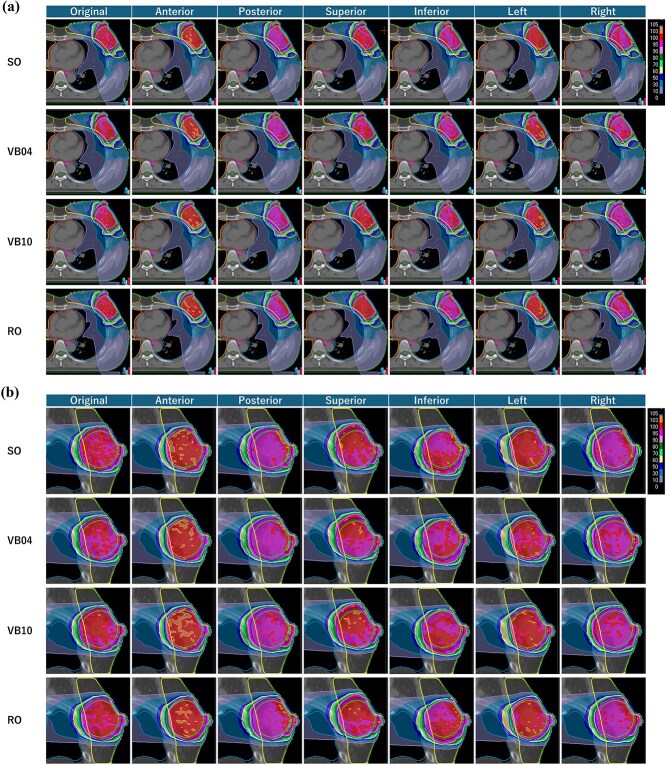
An example of (top) axial and (bottom) sagittal dose distribution when the isocenter is shifted 5 mm in each translational direction. This patient was selected as a representative case, with physique and dose evaluation results that were neither extreme nor atypical in the cohort.

Figure 3 shows a comparison of the perturbed dose of CTV V_95%_ (a: anterior–posterior, b: inferior–superior, c: right–left) and CTV V_105%_ (d: anterior–posterior, e: inferior–superior, f: right–left) among plans due to differences in optimization methods. Figure 4 shows an example of the dose distribution when the isocenter is shifted 5 mm in each translational direction, and Table 3 presents the difference in DVH parameters of CTV, heart, and ipsilateral lung between the original and a 5 mm isocenter shift in each translational direction.

**Table 3:** Absolute difference in dose-volume parameters for the CTV, heart, and ipsilateral lung between the original and a 5 mm isocenter shift in each translational direction (mean and standard deviation)

Variables	Plan	Isocenter shift direction (Mean ± SD)					
		Anterior	Posterior	Superior	Inferior	Left	Right
**CTV**							
V_95%_ (%)	SO	−1.4 ± 1.3	−4.0 ± 3.1	−0.2 ± 0.5	−0.5 ± 0.4	−1.8 ± 2.6	−0.8 ± 1.3
	VB04	−2.3 ± 1.7	1.2 ± 0.9	0.5 ± 0.5	−0.6 ± 0.7	0.0 ± 1.4	−0.3 ± 1.7
	VB10	0.1 ± 0.8	0.3 ± 1.4	0.2 ± 0.4	0.0 ± 0.3	−0.1 ± 1.0	0.3 ± 0.6
	RO	−2.2 ± 1.2	−1.2 ± 0.9	−1.6 ± 0.7	−1.8 ± 0.7	−1.8 ± 1.0	−1.6 ± 1.0
V_105%_ (%)	SO	1.8 ± 1.9	2.7 ± 2.4	0.3 ± 0.3	0.0 ± 0.1	0.6 ± 0.9	0.9 ± 1.4
	VB04	3.3 ± 2.9	2.9 ± 2.5	0.0 ± 0.1	0.1 ± 0.1	1.0 ± 1.5	0.8 ± 1.4
	VB10	10.2 ± 7.3	−1.1 ± 1.5	−0.3 ± 0.9	0.8 ± 1.3	1.4 ± 5.2	1.4 ± 3.2
	RO	0.8 ± 1.2	2.1 ± 1.9	0.3 ± 0.5	0.0 ± 0.1	0.4 ± 0.5	0.6 ± 0.8
D_50%_ (cGy)	SO	35.9 ± 11.5	−38.2 ± 10.9	−5.2 ± 6.7	3.6 ± 6.8	−3.8 ± 32.1	0.9 ± 30.0
	VB04	46.7 ± 9.2	−22.4 ± 18.4	−4.6 ± 5.5	5.5 ± 6.1	5.8 ± 29.6	2.2 ± 30.3
	VB10	50.7 ± 8.6	−45.8 ± 8.2	−8.2 ± 6.0	5.0 ± 5.7	−2.4 ± 32.3	0.5 ± 32.1
	RO	19.6 ± 19.1	−24.4 ± 17.6	−8.4 ± 6.7	0.3 ± 6.0	−3.3 ± 22.9	−4.3 ± 22.5
**Heart**							
D_Mean_ (cGy)	SO	−4.0 ± 6.1	7.2 ± 9.7	4.2 ± 2.4	−3.4 ± 2.1	3.3 ± 8.2	−0.9 ± 5.9
	VB04	−4.2 ± 5.4	8.1 ± 9.5	5.4 ± 2.6	−4.4 ± 2.2	4.0 ± 9.2	−1.0 ± 6.3
	VB10	−3.9 ± 5.6	7.4 ± 9.3	5.0 ± 2.6	−4.0 ± 2.3	3.6 ± 8.4	−1.0 ± 6.1
	RO	−3.7 ± 5.5	6.7 ± 8.7	4.1 ± 2.2	−3.3 ± 2.0	3.4 ± 8.1	−1.0 ± 5.9
**Ipsilateral lung**							
V_30%_ (%)	SO	−1.2 ± 0.5	1.4 ± 0.5	0.4 ± 0.6	−0.4 ± 0.5	0.0 ± 1.6	0.2 ± 1.6
	VB04	−1.3 ± 0.5	1.4 ± 0.5	0.4 ± 0.6	−0.4 ± 0.5	0.0 ± 1.5	0.1 ± 1.5
	VB10	−1.3 ± 0.5	1.4 ± 0.5	0.4 ± 0.6	−0.4 ± 0.5	0.0 ± 1.6	0.2 ± 1.6
	RO	−1.2 ± 0.5	1.3 ± 0.5	0.4 ± 0.5	−0.4 ± 0.5	0.1 ± 1.5	0.1 ± 1.5

Abbreviations: CTV, clinical target volume; SD, standard deviation; SO, standard optimization plan; VB04, virtual bolus plan (*d* = 0.4 g/cm^3^); VB10, virtual bolus plan (*d* = 1.0 g/cm^3^); RO, robust optimization plan.

The lowest CTV V_95%_ (95.3% ± 3.6%) was observed for the SO plan with the isocenter shifted posterior 5 mm, with an average absolute difference of about −4% from the original (no shift) plan. Dose reductions of at least 0.8% were also observed for anterior and lateral shifts. For VB04, CTV V_95%_ coverage increased as the isocenter shifted posteriorly, whereas coverage decreased as the isocenter shifted anteriorly. Changes were small for shifts in other directions. The VB10 plan showed little changes, with a mean change of 0.3% at most, with shifts in any direction. For the RO plan, CTV V_95%_ decreased by approximately 1.0%–2.0% despite the shift direction, but V_95%_ was at least 97% on average.

In the VB10 plan, CTV V_105%_ increased greatly when the isocenter shifted anteriorly, with an average absolute difference of about 10.2%. V_105%_ > 5% was also observed for shifts to the left, right, and inferior directions. For the SO plan, dose increases of ~2% were also observed for anterior and posterior shifts. The V_105%_ increase for the VB04 plan was 3.3% ± 2.9% and 2.9% ± 2.5% for anterior and posterior shifts, respectively, the second largest values after VB10. An increase of ~1% was also observed for shifts to the left or right. The V_105%_ difference of the RO plan was the largest for the posterior shift (2.1% ± 1.9%) but the smallest change compared to other optimization methods.

A characteristic trend was observed between the tumor site (medial or lateral) and isocenter shift in inward and outward directions (Supplementary data). The SO plans showed a larger change in V95% for the inward shift than the outward shift despite the tumor site. In the VB04 plan, the V95% considerably changed in the inward shift compared with the outward shift for a medial tumor. The opposite was true for a lateral tumor. In the VB10 plan, the V_105_% considerably changed in the outward shift compared with the inward shift for a lateral tumor. No trend was observed for the other directions of movement or the tumor site (upper or lower).

For all optimization methods, heart D_mean_ and ipsilateral lung V_30%_ doses showed similar trends, with small differences due to isocenter shift.

**Fig. 5:**
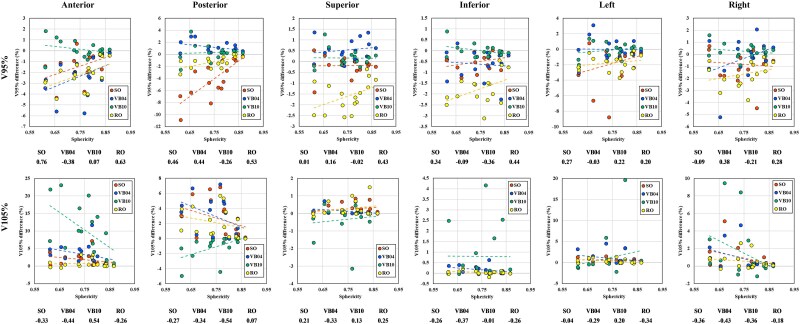
Relationship between the CTV sphericity and dose difference when shifted 5 mm in each direction. Abbreviations: SO, standard optimization plan; VB04, virtual bolus plan (*d* = 0.4 g/cm ^3^); VB10, virtual bolus plan (*d* = 1.0 g/cm ^3^); RO, robust optimization plan.

Figure 5 illustrates the relationship between CTV sphericity and the perturbed dose when shifted 5 mm in each direction. Across all planning approaches, higher sphericity values (closer to 1) were associated with smaller differences in V_95%_ and V_105%_. This trend was particularly pronounced in posterior–anterior shifts.

## DISCUSSION

This study first evaluated DVH parameters of VMAT with different optimization approaches for patients with small physiques and small breasts and subsequently performed perturbed dose evaluation for respiratory motion for those plans. The treatment planning approach using RO resulted in lower OAR dose, better target coverage, and maximum dose than other treatment planning methods. Furthermore, it showed high robustness in isocenter shift simulating respiratory motion and was the most RO method for APBI using VMAT.

With respect to treatment planning, the RO plan showed comparable CTV coverage and maximum dose to the SO plan optimized with the conventional optimization method but had lower PTV_Eval coverage because the RO approach is designed to satisfy the dose constraint in worst-case scenarios and not necessarily to satisfy the coverage in PTV or PTV_Eval regions. The RO plan also had significantly lower OAR doses than the others, possibly due to the difference between optimization methods. APBI-eligible patients are expected to have a better long-term prognosis, and RO plans with reduced irradiation to healthy organs will help mitigate the risk of toxicity and secondary malignancies.

An interesting finding is that the lower PTV_Eval coverage of RO plans, that is, the smaller spread of the high-dose region, was not directly related to the robustness of the plan. Of the 96 perturbed plans (16 patients × 6 direction shifts), the SO, VB04, VB10, and RO plans failed to satisfy the dose constraints of V_95%_ or V_105%_ owing to the isocenter shift in 15, 9, 20, and 3 plans, respectively. This indicates that robust planning was the most robust treatment plan considered in this study.

SO plans developed using conventional optimization methods performed better than VB plans but not as well as RO plans. However, it showed a significant coverage loss at the posterior shift, the condition that reproduced inhalation. RO and VB plans did not cause a decrease in V_95%_, which may be due to the absence of sufficient fluence in the air surrounding the PTV outside the skin surface for the SO plan. Respiratory motion in patients with breast cancer is mainly in anterior–posterior and superior–inferior directions, with reported amplitudes of 3 to 9 mm [11, 26]. Therefore, plans must be robust to these movements. This study revealed that SO plans lack robustness in patients with small breast sizes whose PTVs extend outside the breast, underscoring the necessity of the VB method or RO optimization in such cases. Although SO planning has a simple workflow, it is recommended only for patients with large breasts or when the PTV is entirely within the breast.

The VB04 plan showed favorable treatment planning parameters and high robustness, whereas VB10 showed the worst treatment planning parameters and robustness among the optimization approaches studied. Outlier data were observed in cases of medial tumors with VB10 plans. These occurred because the optimization was performed in CT with a VB, and the final dose calculation and evaluation were performed in CT without a VB. Miyasaka *et al.* and Tang *et al.* reported a similar degradation of dose distribution in PMRT using the VB method [19, 20]. Even APBI with small irradiation and VB volumes caused degradation of dose distribution during the final dose calculation without VB. Lizondo *et al.* recommended low-density VB (−500 HU) to minimize this effect [18], but in this study, high-density VB had a significant impact, so low-density VB is also recommended for APBI. The VB04 plan would be the first choice for APBI treatment planning in institutions without robust planning. Furthermore, optimizing VB thickness and PTV margins may improve robustness to respiratory motion.

The RO plan had a low dependency on shift direction, whereas the other optimization approaches had large variations in the V_95%_ or V_105%_ change in a specific direction. These variations were influenced by the presence of sufficient fluence in the air surrounding the PTV outside the skin surface and optimization effects within the VB. For lateral shifts, the impact of the shift differed based on the tumor site (Supplementary data) probably because the effect of the beam path under the shift changes depending on the tumor site, the dose constraint of the contralateral breast is strict in the case of a medial tumor, and that sufficient fluence is necessary not only on the belly side but also on the lateral side in a lateral tumor. This highlights the importance of tumor location in determining the effect of these fluence in APBI.

The RO plan demonstrated a low MU and larger MCSv than the other optimization methods, indicating a lower level of complexity. By contrast, the VB04 plan exhibited a high MU and low MCSv, making it the most complex plan. Previous studies have reported correlations between complexity metrics and patient-specific quality assurance results [27, 28]. Consequently, simpler plans with higher MCSv values are preferable, as they may reduce uncertainty during beam delivery. In addition, the RO plan had the shortest beam-on time, offering higher throughput and clinical benefits.

This study found that higher CTV sphericity was associated with smaller changes in DVH parameters, indicating greater robustness under isocenter shifts. By contrast, more complex shapes localized the impact of misalignment, reducing plan robustness. Previous studies suggested that sphericity and target shape can serve as quality assessment indices for treatment planning [29, 30]. However, the relationship between CTV sphericity and robustness remains underexplored, warranting further investigation with larger datasets.

To the best of the authors’ knowledge, this is the first study to evaluate the influence of respiratory motion among different optimization approaches for VMAT-APBI. This study focused on patients with small breasts; however, patients with larger breasts relative to the target size may yield different results, and further validation in a cohort including such patients is needed. The main limitation of this study is that the data are based on 16 patients undergoing APBI at a single institution. Although isocenter shifts can account for systematic patient movement, they are inadequate for capturing breast motion, respiratory motion, swelling, and deformity during treatment.

When the patient’s actual breathing is considered, the interplay effects between periodic respiration and MLC motion may alter dose distributions compared with those observed in this study [31]. Interplay effects, which have been extensively studied in lung radiotherapy [32, 33], can average and blur dose distributions. For breast VMAT, limited studies exist; Yamauchi *et al.* evaluated the interplay effect in APBI with VMAT using the SO method, reporting a gamma pass rate of only 84.8% for a 5-mm amplitude [11], which was likely influenced by interplay effects and insufficient fluence outside the skin. This may have resulted from the interplay effect and insufficient fluence outside the skin because of the lack of VB and RO methods. Although this study validated shifts in three orthogonal directions, it did not replicate specific respiratory patterns. However, our results provide a preliminary framework for understanding the relationship between respiratory motion and dose distribution. This framework serves as a conceptual model for estimating dosimetric impacts by considering the amplitude and primary directional tendencies of individual patients’ respiratory motion. Furthermore, the findings emphasize the critical importance of selecting an appropriate optimization method for VMAT-APBI as the choice of method can considerably influence the robustness of the treatment plan against respiratory motion. Further research, including patient-specific respiratory patterns and larger patient cohorts, is required for refining and validating these findings in clinical settings.

An evaluation of the VMAT-APBI optimization approach for patients with a small physique, including breast size, showed that the RO plan provided superior target coverage and maximum dose compared to SO and VB methods. When examining the effects of respiratory motion on the breast, the RO plan also showed the highest robustness, with CTV V_95%_ > 97% and V_105%_ < 2.3% for a 5 mm translational shift. The VB04 plan could be the first choice due to its dosimetric advantages and robustness for institutions where RO is unavailable. However, the VB10 plan had the worst DVH parameters and robustness among the optimization methods. The SO plan had low robustness for patients with PTVs that extend outside the breast because no flush region exists on the outside of the target.

## Supplementary Material

renamed_67526_rraf011

## Human Studies

The study protocol was approved by our institutional review board (22-R009).

## References

[1] National Surgical Adjuvant Breast Bowel Project (NSABP)/radiation therapy oncology group (RTOG) NSABP protocol B-39/RTOG protocol 0413. A randomized phase III study of conventional whole breast irradiation (WBI) versus partial breast irradiation (PBI) for women with stage 0, I, or II breast cancer. Clin Adv Hematol Oncol 2006;4:719–21. 17111558

[2] Vicini, F, Shah, C, Tendulkar, R *et al.* Accelerated partial breast irradiation: an update on published level I evidence. Brachytherapy 2016;15:607–15. 10.1016/j.brachy.2016.06.007. 27475478

[3] Whelan TJ, Julian JA, Berrang TS et al. External beam accelerated partial breast irradiation versus whole breast irradiation after breast conserving surgery in women with ductal carcinoma in situ and node-negative breast cancer (RAPID): a randomised controlled trial. Lancet 2019;394:2165–72. 10.1016/S0140-6736(19)32515-2. 31813635

[4] Livi L, Meattini I, Marrazzo L et al. Accelerated partial breast irradiation using intensity-modulated radiotherapy versus whole breast irradiation: 5-year survival analysis of a phase 3 randomised controlled trial. Eur J Cancer 2015;51:451–63. 10.1016/j.ejca.2014.12.013. 25605582

[5] Quirk S, Grendarova P, Craighead P et al. Results of the ACCEL trial: dosimetry in accelerated partial breast irradiation. Radiother Oncol 2020;147:50–5. 10.1016/j.radonc.2020.03.004. 32224317

[6] Ono Y, Yoshimura M, Hirata K et al. Dosimetric advantages afforded by a new irradiation technique, dynamic WaveArc, used for accelerated partial breast irradiation. Phys Med 2018;48:103–10. 10.1016/j.ejmp.2018.03.015. 29728221

[7] Qiu JJ, Chang Z, Horton JK et al. Dosimetric comparison of 3D conformal, IMRT, and V-MAT techniques for accelerated partial-breast irradiation (APBI). Med Dosim 2014;39:152–8. 10.1016/j.meddos.2013.12.001. 24480375

[8] Marrazzo L, Meattini I, Arilli C et al. Auto-planning for VMAT accelerated partial breast irradiation. Radiother Oncol 2019; 132:85–92. 10.1016/j.radonc.2018.11.006. 30825975

[9] Marrazzo L, Meattini I, Simontacchi G et al. Updates on the APBI-IMRT-Florence trial (NCT02104895) technique: from the intensity modulated radiation therapy trial to the volumetric modulated arc therapy clinical practice. Pract Radiat Oncol 2023;13:e28–34. 10.1016/j.prro.2022.05.010. 35659597

[10] Quirk S, Grendarova P, Roumeliotis M. Five-field IMRT class solutions and dosimetric planning guidelines for implementing accelerated partial breast irradiation. Pract Radiat Oncol 2018;8:e99–107. 10.1016/j.prro.2017.09.009. 29141779

[11] Yamauchi R, Mizuno N, Itazawa T, Kawamori J. The influence of respiratory motion on dose distribution in accelerated partial breast irradiation using volumetric modulated arc therapy. Phys Med 2020;80:23–33. 10.1016/j.ejmp.2020.09.024. 33075732

[12] Mitsumori M, Hiraoka M. Current status of accelerated partial breast irradiation. Breast Cancer 2008;15:101–7. 10.1007/s12282-007-0012-1. 18224403

[13] Kosaka Y, Mitsumori M, Yamauchi C et al. Feasibility of accelerated partial breast irradiation using three-dimensional conformal radiation therapy for Japanese women: a theoretical plan using six patients' CT data. Breast Cancer 2008;15:108–14. 10.1007/s12282-007-0013-0. 18224404

[14] Giorgia N, Antonella F, Alessandro C et al. Planning strategies in volumetric modulated are therapy for breast. Med Phys 2011;38:4025–31. 10.1118/1.3598442. 21859000

[15] He Y, Chen S, Gao X et al. Robustness of VMAT to setup errors in postmastectomy radiotherapy of left-sided breast cancer: impact of bolus thickness. PLoS One 2023;18:e0280456. 10.1371/journal.pone.0280456. 36693073 PMC9873183

[16] Rossi M, Boman E, Kapanen M. Optimal selection of optimization bolus thickness in planning of VMAT breast radiotherapy treatments. Med Dosim 2019;44:266–73. 10.1016/j.meddos.2018.10.001. 30389413

[17] Tyran M, Tallet A, Resbeut M et al. Safety and benefit of using a virtual bolus during treatment planning for breast cancer treated with arc therapy. J Appl Clin Med Phys 2018;19:463–72. 10.1002/acm2.12398. 29959819 PMC6123145

[18] Lizondo M, Latorre-Musoll A, Ribas M et al. Pseudo skin flash on VMAT in breast radiotherapy: optimization of virtual bolus thickness and HU values. Phys Med 2019;63:56–62. 10.1016/j.ejmp.2019.05.010. 31221409

[19] Tang R, Li A, Li Y et al. Dosimetric comparison of two dose expansion methods in intensity modulated radiotherapy for breast cancer. Radiat Oncol 2023;18:23. 10.1186/s13014-023-02217-4. 36737788 PMC9898932

[20] Miyasaka Y, Ono T, Chai H et al. A robust treatment planning approach for chest motion in postmastectomy chest wall intensity modulated radiation therapy. J Appl Clin Med Phys 2024;25:e14217. 10.1002/acm2.14217. 38018758 PMC10795451

[21] Fredriksson A, Forsgren A, Hardemark B. Minimax optimization for handling range and setup uncertainties in proton therapy. Med Phys 2011;38:1672–84. 10.1118/1.3556559. 21520880

[22] White J, Tai A, Arthur D et al. Breast Cancer Atlas for Radiation Therapy Planning: Consensus Definitions*.* http://www.rtog.org/ (7 Dec 2024, date last accessed).

[23] Shaitelman SF, Anderson BM, Arthur DW et al. Partial breast irradiation for patients with early-stage invasive breast cancer or ductal carcinoma in situ: an ASTRO clinical practice guideline. Pract Radiat Oncol 2024;14:112–32. 10.1016/j.prro.2023.11.001. 37977261

[24] Cavinato S, Scaggion A, Paiusco M. Technical note: a software tool to extract complexity metrics from radiotherapy treatment plans. Med Phys 2024;51:8602–12. 10.1002/mp.17365. 39186793

[25] Kanda Y . Investigation of the freely available easy-to-use software 'EZR' for medical statistics. Bone Marrow Transplant 2013;48:452–8. 10.1038/bmt.2012.244. 23208313 PMC3590441

[26] Kinoshita R, Shimizu S, Taguchi H et al. Three-dimensional intrafractional motion of breast during tangential breast irradiation monitored with high-sampling frequency using a real-time tumor-tracking radiotherapy system. Int J Radiat Oncol Biol Phys 2008;70:931–4. 10.1016/j.ijrobp.2007.10.003. 18164868

[27] Masi L, Doro R, Favuzza V et al. Impact of plan parameters on the dosimetric accuracy of volumetric modulated arc therapy. Med Phys 2013;40:071718. 10.1118/1.4810969. 23822422

[28] Chiavassa S, Bessieres I, Edouard M et al. Complexity metrics for IMRT and VMAT plans: a review of current literature and applications. Br J Radiol 2019;92:20190270. 10.1259/bjr.20190270. 31295002 PMC6774599

[29] Chagas , Saraiva CW, Cardoso SC, Groppo DP et al. Gamma knife radiosurgery for vestibular schwannomas: evaluation of planning using the sphericity degree of the target volume. PLoS One 2020;15:e0225638. 10.1371/journal.pone.0225638. 31923229 PMC6953829

[30] McGurk R, Smith V, Price M. Evaluating IMRT plan deliverability via PTV shape and MLC motion. Med Phys 2014;41:459. 10.1118/1.4889281.

[31] Bortfeld T, Jiang SB, Rietzel E. Effects of motion on the total dose distribution. Semin Radiat Oncol 2004;14:41–51. 10.1053/j.semradonc.2003.10.011. 14752732

[32] Chen H, Wu A, Brandner ED et al. Dosimetric evaluations of the interplay effect in respiratory-gated intensity-modulated radiation therapy. Med Phys 2009;36:893–903. 10.1118/1.3070542. 19378749

[33] Netherton T, Li Y, Nitsch P et al. Interplay effect on a 6-MV flattening-filter-free linear accelerator with high dose rate and fast multi-leaf collimator motion treating breast and lung phantoms. Med Phys 2018;45:2369–76. 10.1002/mp.12899. 29611210

