# Supplementary material for: A robust planning approach for respiratory motion in accelerated partial breast irradiation using volumetric modulated arc therapy
Source: J Radiat Res. 2025 Mar 10;66(2):185–95. doi: 10.1093/jrr/rraf011 (PMC11932346; doi:10.1093/jrr/rraf011)
Supplement: renamed_67526_rraf011 [file renamed_67526_rraf011.docx]

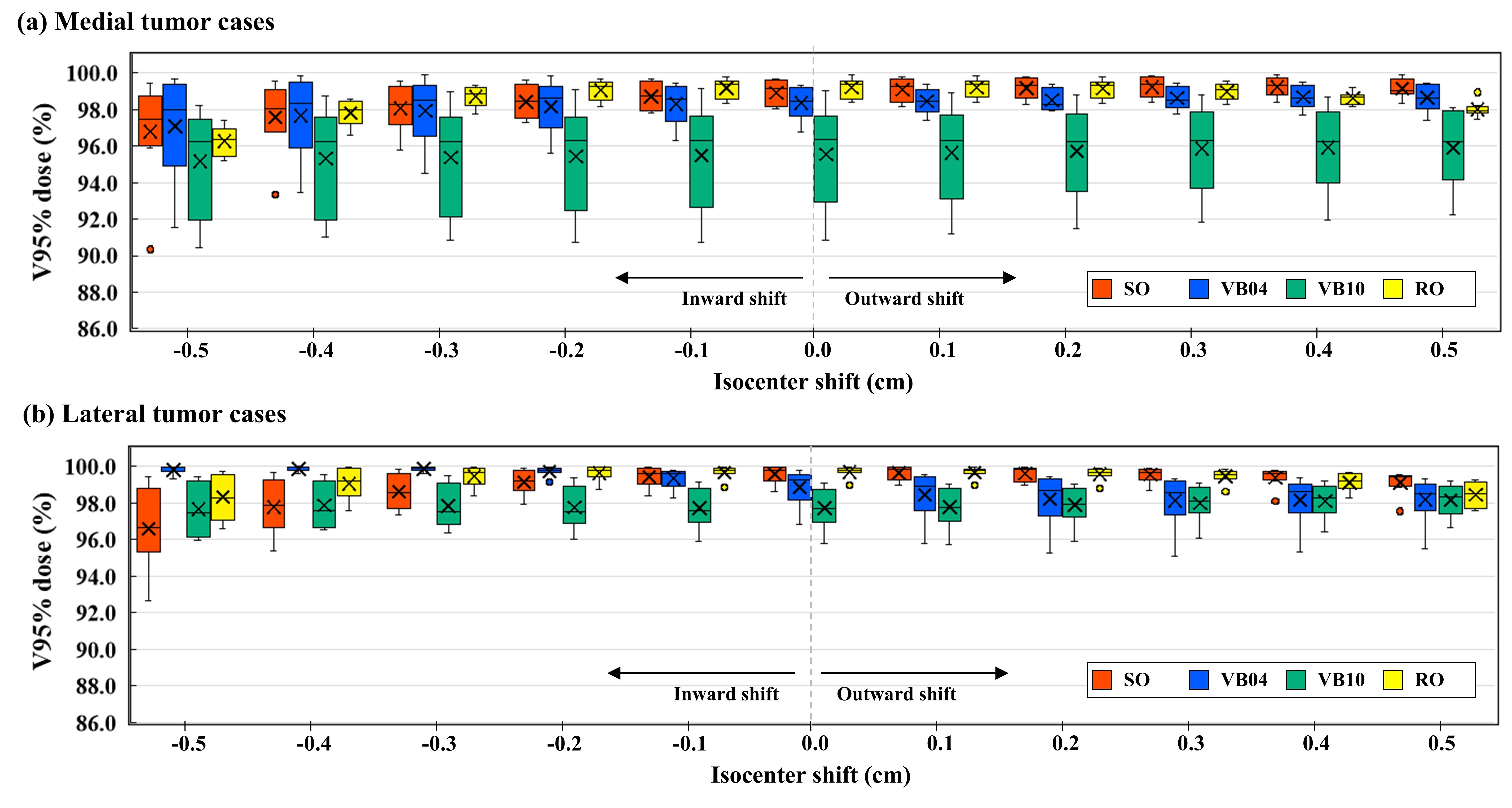


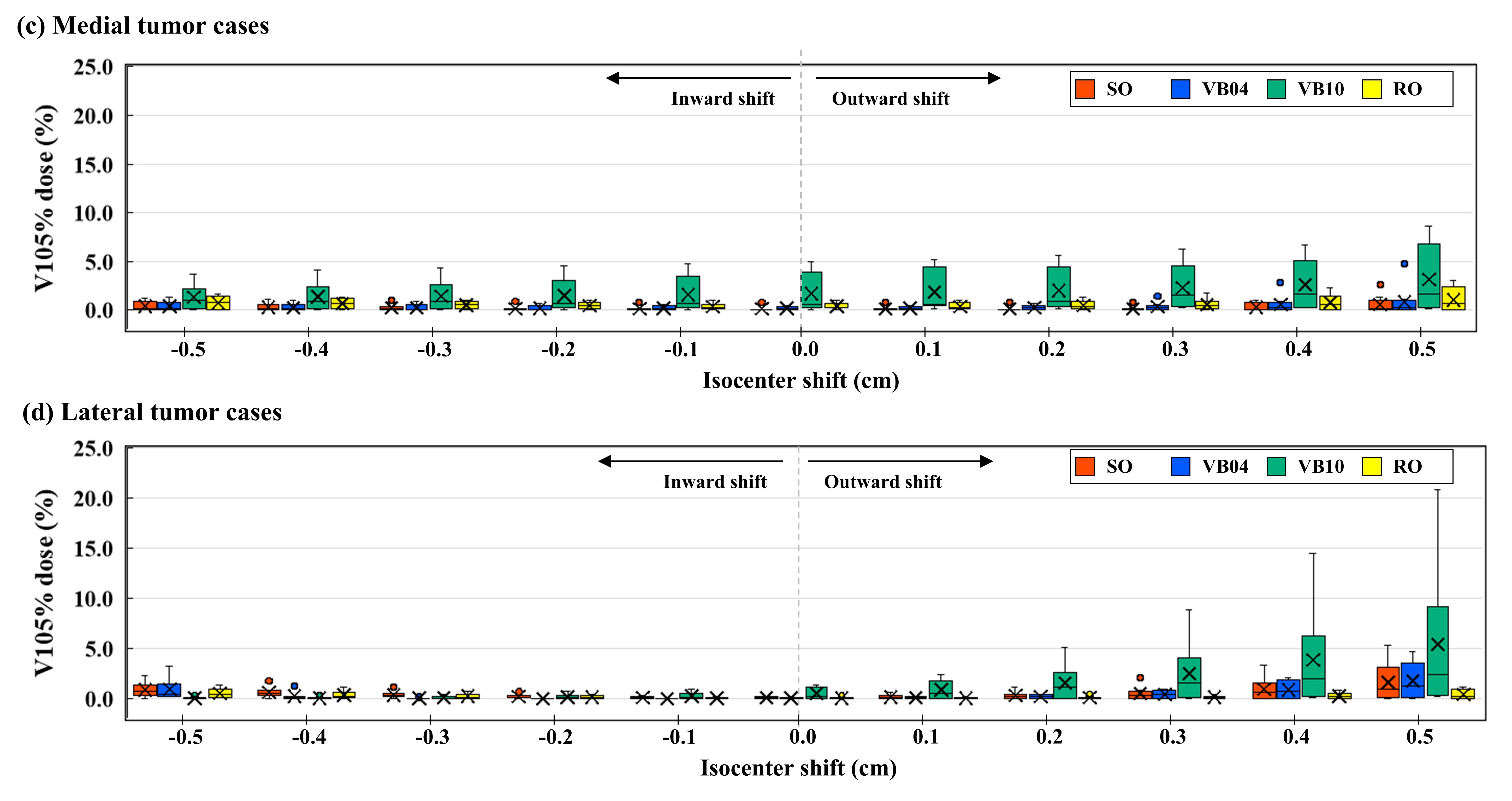


**Supplementary file 1**. Comparison of the box plots of CTV V95% and V105% for four different optimization methods in the shift in the medial-lateral direction when classified by tumor location (medial or lateral).
